# Abnormal Liver Function Test in Patients Infected with Coronavirus (SARS-CoV-2): A Retrospective Single-Center Study from Spain

**DOI:** 10.3390/jcm10051039

**Published:** 2021-03-03

**Authors:** Raquel Benedé-Ubieto, Olga Estévez-Vázquez, Vicente Flores-Perojo, Ricardo U. Macías-Rodríguez, Astrid Ruiz-Margáin, Eduardo Martínez-Naves, José R. Regueiro, Matías A. Ávila, Christian Trautwein, Rafael Bañares, Jaume Bosch, Francisco Javier Cubero, Yulia A. Nevzorova

**Affiliations:** 1 Department of Immunology, Ophthalmology & ENT, Complutense University School of Medicine, 28040 Madrid, Spain; rabenede@ucm.es (R.B.-U.); olgaeste@ucm.es (O.E.-V.); emnaves@ucm.es (E.M.-N.); regueiro@ucm.es (J.R.R.); 2 Department of Genetics, Physiology and Microbiology, Faculty of Biology, Complutense University, 28040 Madrid, Spain; 3 iBroker Global Markets, SV, SA, 28033 Madrid, Spain; vicente@ibroker.com; 4 Department of Gastroenterology, Instituto Nacional de Ciencias Médicas y Nutrición Salvador Zubirán, Mexico City 14080, Mexico; ricardomacro@yahoo.com.mx (R.U.M.-R.); ruizm.astrid@gmail.com (A.R.-M.); 5 12 de Octubre Health Research Institute (imas12), 28041 Madrid, Spain; 6 Hepatology Program, CIMA, University of Navarra, 31009 Pamplona, Spain; maavila@unav.es; 7 Instituto de Investigaciones Sanitarias de Navarra IdiSNA, 31008 Pamplona, Spain; 8 CIBERehd, Instituto de Salud Carlos III, 28220 Madrid, Spain; rafael.banares@salud.madrid.org (R.B.); jaume.bosch@idibaps.org (J.B.); 9 Department of Internal Medicine III, University Hospital RWTH Aachen, 52074 Aachen, Germany; ctrautwein@ukaachen.de; 10 Health Research Institute Gregorio Marañón (IiSGM), 28007 Madrid, Spain; 11 Barcelona Hepatic Haemodynamic Laboratory, Liver Unit, Institute of Digestive and Metabolic Diseases, August Pi I Sunyer Institute of Biomedical Research, Hospital Clinic, 08036 Barcelona, Spain; 12 Inselspital, Bern University, 3010 Bern, Switzerland

**Keywords:** SARS-CoV-2, liver injury, pandemic, AST/ALT ratio, ferritin

## Abstract

The outbreak of the novel coronavirus SARS-CoV-2 epidemic has rapidly spread and still poses a serious threat to healthcare systems worldwide. In the present study, electronic medical records containing clinical indicators related to liver injury in 799 COVID-19-confirmed patients admitted to a hospital in Madrid (Spain) were extracted and analyzed. Correlation between liver injury and disease outcome was also evaluated. Serum levels of Alanine aminotransferase (ALT), Aspartate aminotransferase (AST), Gamma-glutamyltransferase (GGT), Alkaline phosphatase (ALP), Lactate dehydrogenase (LDH) and AST/ALT ratio were elevated above the Upper Limit of Normal (ULN) in 25.73%, 49.17%, 34.62%, 24.21%, 55.84% and 75% of patients, respectively. Interestingly, significant positive correlation between LDH levels and the AST/ALT ratio with disease outcome was found. Our data showed that SARS-CoV-2 virus infection leads to mild, but significant changes in serum markers of liver injury. The upregulated LDH levels as well as AST/ALT ratios upon admission may be used as additional diagnostic characteristic for COVID-19 patients.

## 1. Introduction

The outbreak of a novel enveloped RNA β coronavirus, named severe acute respiratory syndrome coronavirus-2 (SARS-CoV-2), is a major challenge for health systems worldwide from the beginning of 2020. As a result of its rampant spread and clinical severity, the World Health Organization (WHO) officially recognized the coronavirus disease (COVID-19) as a pandemic on 11 March, 2020. By February 2021, the virus had already infected over 114 million people worldwide, causing more than 2.5 millions deaths and at least 69,142 deaths in Spain alone.

A remarkable amount of clinical studies were published in order to elucidate the clinical manifestations of SARS-CoV-2 infection. Apart from asymptomatic or paucisymptomatic forms, and severe viral pneumonia with respiratory failure, COVID-19 disease prognosis is influenced by indirect effects on body-wide organs via the angiotensin-converting enzyme-2 (ACE-2) receptor, including heart and renal failure, damage to the central nervous system (CNS) and to the gastrointestinal tract [1,2].

Specifically, in the liver, the effect of COVID-19 has been evaluated in relatively few studies, which focused on alterations in liver histopathology and biochemistry during the course of infection in patients who succumbed to the virus [3,4,5,6,7,8]. Several up-to-date studies have attempted to evaluate parameters related to liver injury in American and Asian cohorts [3,8,9,10,11], as well as in European cohorts [12,13,14,15]. Yet liver disease in the context of SARS-CoV-2 infection remains largely unknown, thus minimizing the understanding of abnormal liver function in terms of the overall outcome.

Therefore, we performed a retrospective single-center analysis of laboratory indicators of liver injury upon admission in a large cohort of COVID-19-confirmed patients admitted to the Hospital Universitario 12 de Octubre in Madrid (Spain) during the current outbreak.

## 2. Materials and Methods

### 2.1. Study Design and Patients

This retrospective single-center study was performed from 25 February 2020 to 23 April 2020 in patients admitted to the Hospital Universitario 12 de Octubre in Madrid. A total of 799 were confirmed cases of SARS-Cov-2. Demographic and clinical parameters of interest were collected from electronic medical records (Table 1). Patients were followed-up until death, hospital discharge or the end of the observation period (23 April 2020). Personal data complied with the Law 14/2007 of 3 July on Biomedical Research (Spain), as well as the EU general data protection regulation (GDPR) 2016/679 of the European Parliament and of the Council of 27 April 2016, and the Organic Law 3/2018, of 5 December on Data Protection and Guarantee of Digital Rights (Spain).

Confirmed diagnosis of SARS-CoV-2 was based on a positive quantitative real-time (RT) reverse transcriptase PCR (qRT-PCR) assay performed on oropharyngeal swab (Thermofisher TaqPath™ 1-Step RT-qPCR Master Mix, CG).

Clinical characteristics data included oxygen saturation levels (SO_2_) by pulse oximetry (%) and blood chemical analyses which were routinely measured using standard methods on the first day of hospitalization before administration of any therapy. Laboratory evaluations consisted of alanine aminotransferase (ALT), aspartate aminotransferase (AST), gamma-glutamyltransferase (GGT), alkaline phosphatase (ALP) and lactate dehydrogenase (LDH), ferritin, fibrinogen, prothrombin time (PT (s)) and D-dimers (DD).

Abnormality in test parameters was defined as the elevation of ALT > 41 U/L, AST > 38 U/L, GGT > 58 U/L, ALP > 96 U/L, LDH > 333 U/L, ferritin> 200 ng/mL for women and > 500 ng/mL for men, fibrinogen > 400 mg/dL, PT > 14s, DD > 250 µg/L and SO_2_ level < 90%.

### 2.2. Statistical Analysis

Analyses were performed using IBM SPSS v25 (IBM Corp. Armonk, NY, USA). Data were expressed as means ± standard deviation (SD). Comparisons between groups were performed using *T*-test for independent samples. *p*-values for significance are indicated as follows: *: *p* < 0.05; **: *p* < 0.01; ***: *p* < 0.001. Distribution plots and correlations were analyzed. The relation between liver function tests (LFTs) and variables such as age, SO_2_ and coagulation parameters were analyzed. Recently were considered as dependent variables, while the rest were considered as independent or predictor variables. Independent and dependent variables were correlated using lineal regression. Correlation coefficient (*R*^2^) and cases (%) represented were studied in detail.

Multivariant analysis between LFTs, age, gender and outcome was performed. Interactions between those independent factors and LFTs were analyzed in detail via two-way analysis of variance (ANOVA) two-way. *p*-value (*p* < 0.05) indicated real interaction between determined factors for each LFTs marker.

## 3. Results

Of all participants enrolled in this study, 362 (45.3%) were women and 437 (54.7%) were men *(*Table 1). Patients were predominantly middle-aged with a mean age of 64.11 ± 16.92 years of age. Of the 799 patients, 140 (17.5%) patients died and 659 (82.5%) survived *(*Table 1).

For most of the 799 patients, mean values for liver tests (LT) were normal as indicated by levels of ALT (38.07 ± 39.61 U/L), AST (43.57 ± 35.35 U/L), GGT (69.42 ± 85.11 U/L), ALP (87.32 ± 67.91 U/L) or slightly increased in the case of LDH (369.58 ± 159.08 U/L) (Table 2). Elevated ALT, AST, GGT and ALP levels were detected in 25.73%, 49.17%, 34.62% and 24.21% of patients, respectively. Interestingly, LDH levels were raised in 55.8% of patients.

**Figure 1:**
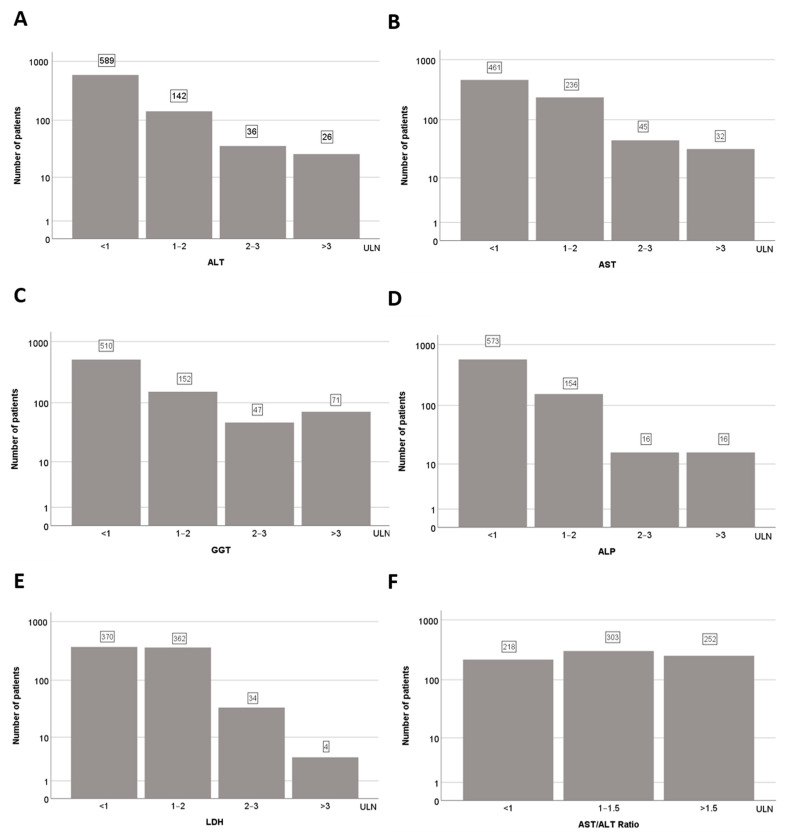
Liver function tests (LFTs) in patients with COVID-19 at admission. Number of patients within and above the upper limits of normal (ULN) for (**A**) ALT, (**B**) AST, (**C**) GGT, (**D**) ALP, (**E**) LDH and (**F**) AST/ALT ratio.

In most patients, elevations of liver parameters were within 2× the upper limit of normal (ULN) (Table 2 and Figure 1A–E). For example, 362 patients had LDH within 2 × ULN, 34 within 2–3 ULN and only 4 above 3 × ULN (Figure 1E). Moreover, the majority of patients (75%) had higher AST than ALT values, with a De Ritis ratio (AST/ALT) of 1.41 ± 0.91 (Table 2 and Figure 1F).

**Figure 2:**
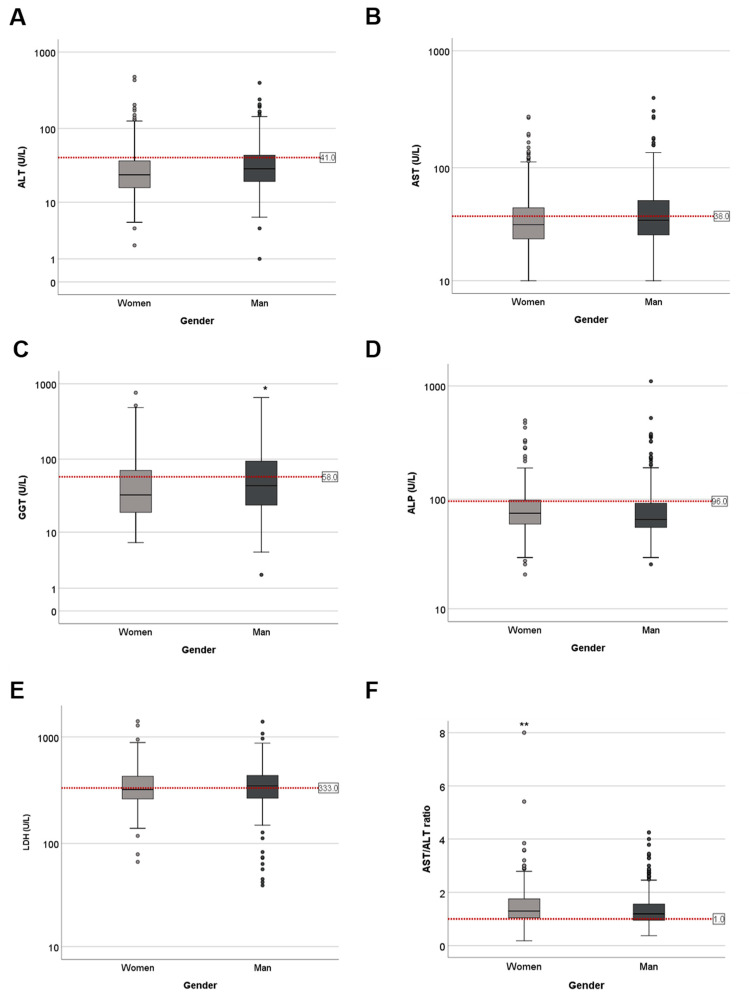
Gender influence on LFTs changes in COVID-19 positive patients at admission. Serum levels of (**A**) ALT. Women = 35.55 ± 2.39; men = 40.04 ± 1.85 (**B**) AST. Women = 41.67 ± 1.83; men = 45.53 ± 1.91 (**C**) GGT. Women = 61.57 ± 4.59; men = 76.53 ± 4.37 (**D**) ALP. Women = 88.59 ± 3.16; men = 86.00 ± 3.79 (**E**) LDH. Women = 363.85 ± 8.9; men = 369.88 ± 7.77 and (**F**) AST/ALT. Women = 1.65 ± 0.36; men = 1.55 ± 0.69. Ratios in women (light grey) and men (dark grey); (**-* *p* < 0.01–0.05).

Men presented higher level of GGT (Figure 2C); women had higher De Ritis ratio levels (Figure 2F). However, ALT, AST, ALP and LDH values did not show any difference due to gender (Figure 2).

**Figure 3:**
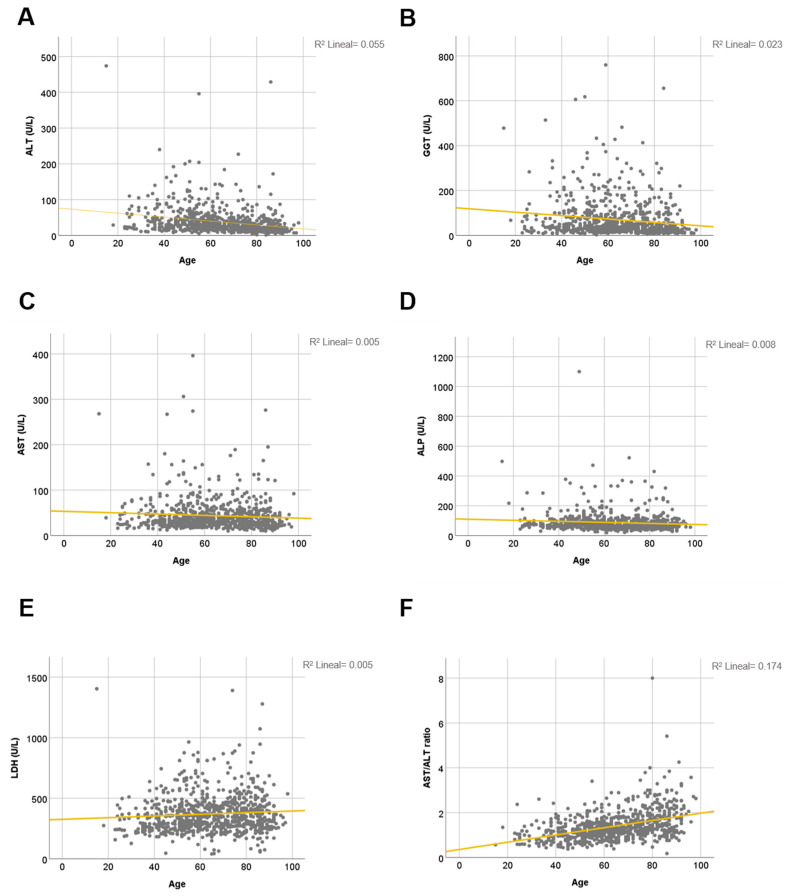
Correlation between liver biochemical parameters at admission and age in COVID-19-confirmed patients. (**A**) ALT; Pearson coefficient = −0.234; F value = 45.973; *p* < 0.0001. (**B**) AST; Pearson coefficient = −0.072; F value = 3.994; *p* = 0.046. (**C**) GGT; Pearson coefficient = −0.051: F value = 18.270; *p* < 0.0001. (**D**) ALP; Pearson coefficient = −0.088; F value = 5.584; *p* = 0.016. (**E**) LDH; Pearson coefficient = 0.073; F value = 4.416; *p* = 0.042. (**F**) AST/ALT ratio; Pearson coefficient = 0.329; F value = 93.633; *p* < 0.0001. *R*^2^ calculations appear on the upper right side of each graph. Age interval, 10 years.

The correlations between age of the patient and serum markers of liver injury were mild (Table 2 and Figure 3A–E). Only AST/ALT ratio had a significant positive correlation with age in 32.9% of cases and on average was 1.87–2.05 in patients over 80 years (Table 2 and Table 3 and Figure 3F).

**Figure 4:**
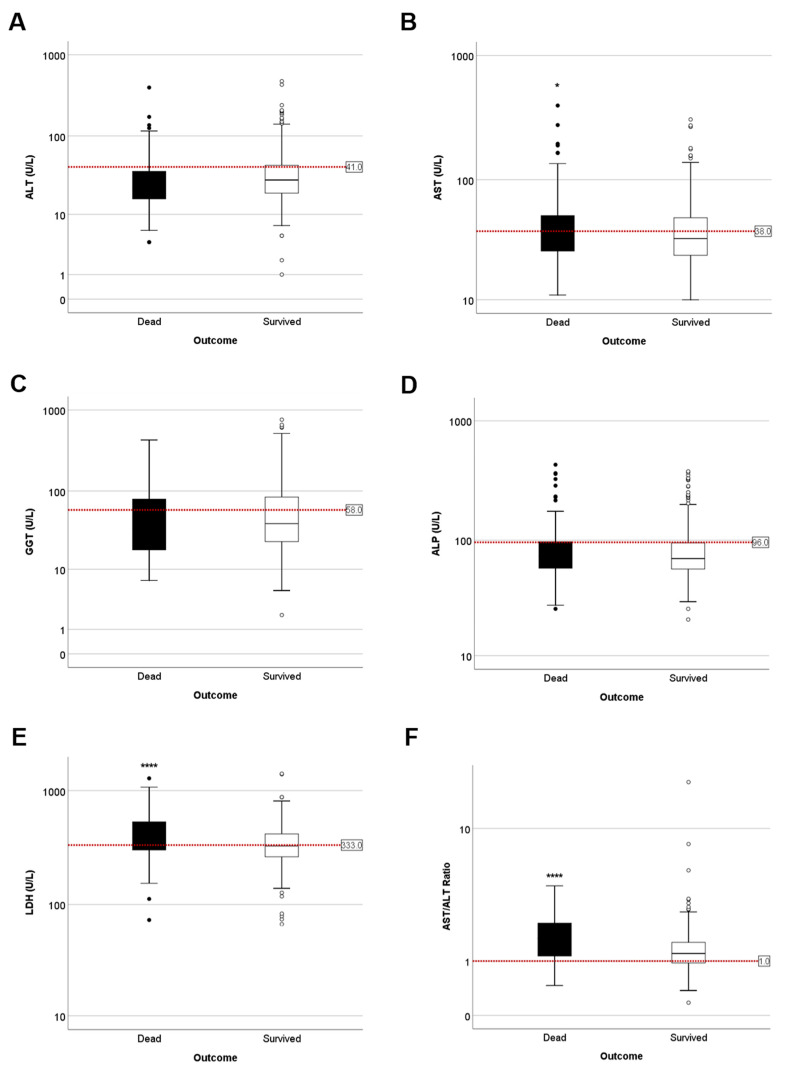
Disease outcome for COVID-19 patients based on LFTs parameters at admission. (**A**) ALT. Dead = 33.58 ± 3.88; survived = 38.88 ± 1.60 (**B**) AST. Dead = 51.51 ± 4.71; survived = 42.24 ± 1.29 (**C**) GGT. Dead = 64.43 ± 6.87; survived = 70.80 ± 3.56 (**D**) ALP. Dead = 91.53 ± 5.98; survived = 86.31 ± 2.77 (**E**) LDH. Dead = 439.73 ± 19.17; survived = 352.73 ± 5.72 and (**F**) AST/ALT. Dead = 1.80 ± 0.79; survived = 1.33 ± 0.91. Ratio was calculated and divided between non-survivors (deceased, black) and survivors (white); (**** *p* < 0.0001).

Additionally, we compared liver parameters between COVID-19 survivors and non-survivors (Table 2). No obvious difference was found in ALT, GGT and ALP levels between COVID19 survivors and non-survivors (Table 2 and Figure 4A–D). Remarkably, levels of AST and LDH were higher in patients with bad prognosis versus patients who survived: 51.51 ± 4.71 vs. 42.24 ± 1.29 and 439.72 ± 19.17 vs. 352.73 ± 5.72, respectively (Table 2 and Figure 4E). Interestingly, patients who died had significantly higher AST/ALT ratios upon admission when compared with patients who survived (1.80 ± 0.69 vs. 1.33 ± 0.36) (Figure 4F).

Clinical characteristics of the patients and LFTs were included in the multivariate analysis (Supplementary Table S1), which showed that gender, survival and age were significantly associated with AST and LDH; and only gender and survival had significant interaction with AST/ALT ratio.

In patients with severe COVID-19, pneumonia, respiratory failure, systemic inflammation and thrombotic complications are the most frequent [16]. Therefore, next we investigated whether AST, LDH levels and De Ritis ratio correlate with disease severity. We analyzed these three liver-related parameters which were mainly upregulated in our patients and compared them with laboratory markers associated with COVID-19 complications.

**Figure 5:**
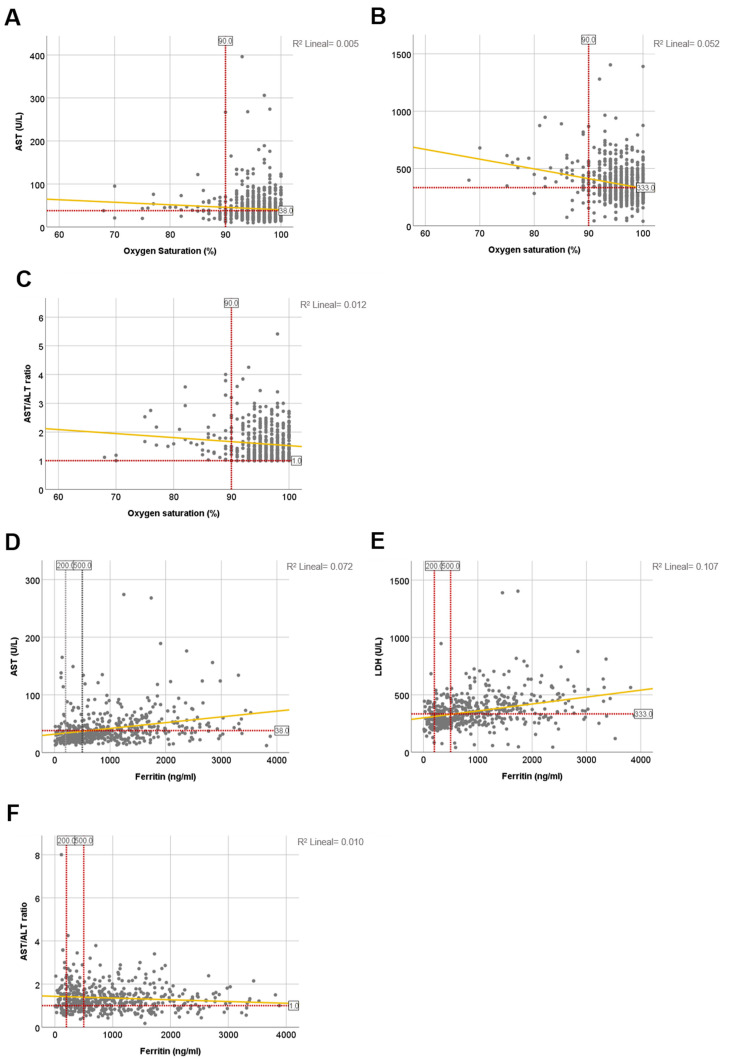
The correlation between biochemical LFTs with SO_2_ and ferritin, and main coagulogram parameters in COVID −19 patients. (**A**) AST and SO_2_; Pearson coefficient = −0.072; F value = 3.723; *p* = 0.054. (**B**) LDH and SO_2_; Pearson coefficient = −0.228; F value = 38.924; *p* < 0.0001. (**C**) AST/ALT ratio and SO_2_; Pearson coefficient = −0.106; F value = 8.087; *p* < 0.005. (**D**) AST and ferritin; Pearson coefficient = 0.434; F value = 121.577; *p* < 0.0001. (**E**) LDH and ferritin; Pearson coefficient = 0.357; F value = 75.464; *p* < 0.0001. (**F**) AST/ALT ratio and ferritin; Pearson coefficient = −0.079; F value = 3.245; *p* = 0.072. *R*^2^ calculations appear on the upper right side of each graph.

COVID-19 primarily attacks the respiratory system leading to pneumonia and oxygen deprivation [17]. Hence, upon admission only 7% of patients demonstrated signs of hypoxia with oxygen saturation (SO_2_) level <90%. No significant correlation between SO_2_ and liver-related parameters was found in our study *(*Table 3 and Table 4 and Figure 5A–C).

Several studies reported that COVID-19 patients have elevated levels of ferritin, and that this marker is strongly associated with the development of systemic inflammation in COVID-19 patients [18]. Indeed, ferritin was elevated in 81.15% of the patients upon admission time *(*Table 3), and was significantly higher in patients with bad prognosis. However, we found positive correlation between ferritin and AST in 43.3% of the cases; and between ferritin and LDH in 35.5% of patients *(*Figure 5D–F and Table 5).

**Figure 6:**
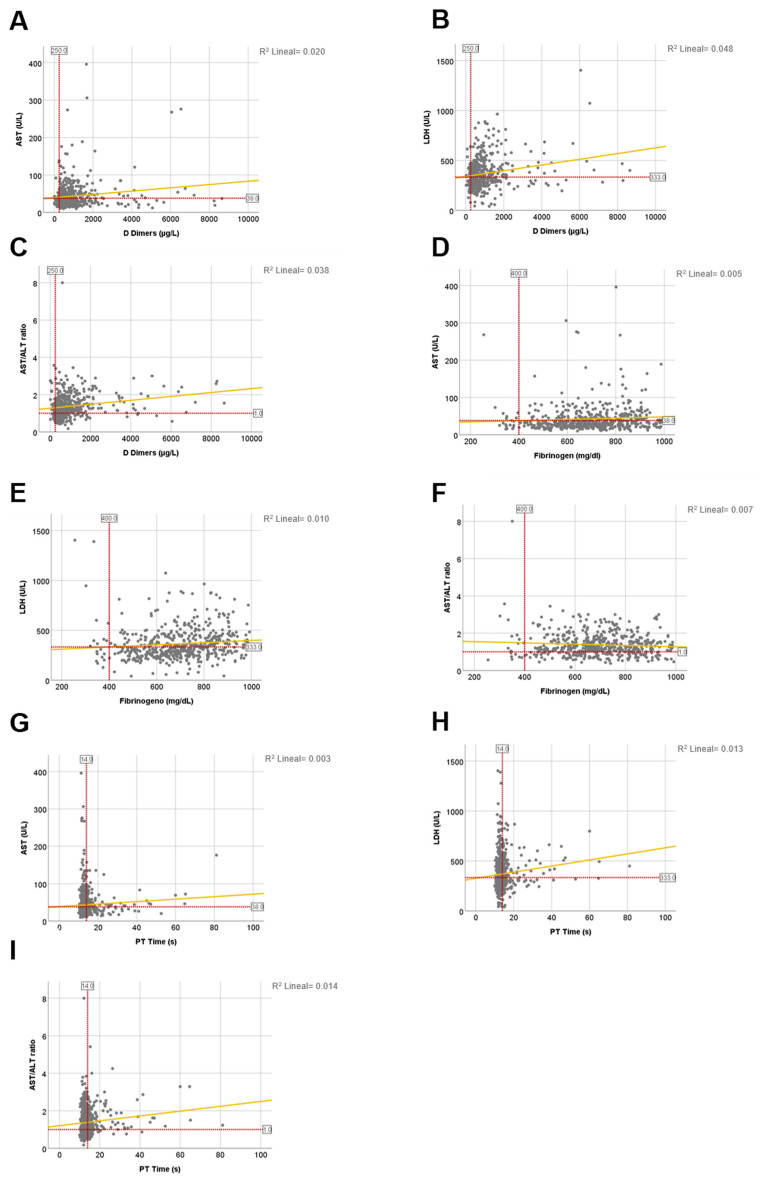
The correlation between biochemical LFTs and main coagulogram parameters in COVID-19 patients. (**A**) AST and DD; Pearson coefficient = 0.076; F value = 2.889; *p* = 0.09. (**B**) LDH and DD; Pearson coefficient = 0.365; F value = 74.716; *p* < 0.0001. (**C**) AST/ALT ratio and DD; Pearson coefficient = 0.041; F value = 0.836; *p* = 0.361. (**D**) AST and fibrinogen; Pearson coefficient = 0.104; F value = 6.243; *p* = 0.013. (**E**) LDH and fibrinogen; Pearson coefficient = 0.152; F value = 13.307; *p* < 0.0001. (**F**) AST/ALT ratio and fibrinogen; Pearson coefficient = −0.116; F value = 7.808; *p* = 0.005. (**G**) AST and PT time; Pearson coefficient = 0.008; F value = 0.044; *p* = 0.833. (**H**) LDH and PT time; Pearson coefficient = 0.035; F value = 0.934; *p* = 0.334. (**I**) AST/ALT ratio and PT time; Pearson coefficient = 2.131; F value = 6.243; *p* = 0.143. *R*^2^ calculations appear on the upper right side of each graph.

The coagulopathy accompanying COVID-19 has been associated with extremely elevated DD and fibrinogen levels as well as modest prolongations of prothrombin time (PT) [19]. In fact, in our study we found increased fibrinogen in 96.61%, prolongation of PT time in 69.64% and elevation of DD in 90.98% of the admitted patients (Table 4). Moreover, DD were dramatically higher (*p* = 0.016) in patients with bad prognosis versus patients who survived. However, COVID-associated coagulopathy was relatively weakly correlated with AST, LDH or De Ritis ratio (Figure 6A–I and Table 6). Such as, in 36.5% of the cases the LDH level had significant positive correlation with elevated DD, and with fibrinogen in 15.2% of the cases. AST showed significant correlation with elevated fibrinogen in 10.4% of the patients (Table 6).

## 4. Discussion

To what extent liver damage is relevant to the novel SARS-CoV-2 pandemic remains still unclear. In the present study, we described clinical characteristics of 799 patients who were admitted to the Hospital Universitario 12 de Octubre in Madrid (Spain), with COVID-19 symptoms and who tested positive for COVID-19. We used the values of ALT, AST, ALP, GGT and LDH as the predominant screening examinations of the functional status of the liver.

Here we report that upon admission, ALT, GGT and ALP were significantly elevated in approximately 25–35% of COVID-19-confirmed cases. While 49.17% of patients presented elevations in AST levels, nearly 56% had increased LDH values. A few previous clinical studies based on single or multiple centers have already shown that COVID-19 patients have signs of liver injury [5] (Supplementary Table S1). Concomitant with our findings, two studies from Wuhan showed that approximately 35–53% of COVID-19 patients had elevated AST [3,20]. In a larger American cohort that included 5700 patients, 59% and 39% showed AST and ALT values above ULN, respectively [10]. In agreement with our data, two independent studies from Northern Italy [12] and Austria [15] demonstrated upregulation of AST in 44% and 42% of admitted patients with SARS-CoV-2, respectively. In contrast, fewer patients (only 16% and 20%) had abnormal AST levels in the studies from Zhejiang Province [4] and Rome [13]. The increase in LDH was detected in 27% COVID-patients of Zhejiang Province [4] and in 73% patients in Wuhan [4,21] (Supplementary Table S2).

These findings are worth discussing since the SARS-CoV-2 virus should be potentially regarded as hepatotropic. Angiotensin converting enzyme 2 (ACE2) was identified as a functional receptor for SARS-CoV-2, and ACE2 expression is high in lung, heart, ileum, kidney and bladder [2]. However, the expression level of ACE2 in liver tissue was only approximately 0.31% and the specific expression of ACE2 in bile duct epithelial cells was 20 times higher than that in hepatocytes [22,23]. Thus, potential damage to cholangiocytes by SARS-CoV-2 may lead to profound and unexpected consequences in the liver. Hence, despite the presence of ACE2 in cholangiocytes, more patients developed raised levels of transaminases [23]. On the other hand, there exist wide discrepancies between organ symptomatology and ACE2 expression levels. For instance, ACE2 expression in the respiratory tract is only moderate compared with that in intestinal epithelia, but respiratory symptomatology is substantially more severe than intestinal [24,25].

Nonetheless, pathological analysis of liver tissue from a patient who died from COVID-19 showed that viral inclusions were not observed in the liver [26]. Any reported [27] ‘spiked’ inclusions and degenerate ‘corona-like’ particles in hepatocytes were not confirmed by PCR testing for viral nucleic acids [28].

A very likely explanation of the mechanisms leading to liver dysfunction during COVID-19 refers not to the direct cytopathic effects of the virus, but rather to the generalized stress due to the multi-organic character of the infection, which is accompanied by immune injury, systemic inflammatory response syndrome (SIRS) and a stormy release of cytokines that can cause liver injury per se [23,29,30].

One of the clinical characteristics of COVID-19-derived SIRS is “hyperferritinemic syndrome” and high serum ferritin level [31]. Indeed, 81% of patients in our study had elevated levels of ferritin upon admission. Moreover, ferritin levels were significantly higher in patients who subsequently died. Hence, 43.3% of the patients had significant positive correlation between levels of ferritin and AST, and 35.7% between ferritin and LDH levels.

Another important finding of the present study was the elevated LDH levels found in patients with COVID-19 infection already at the time of admission, and its positive correlation with disease outcome. The increase of LDH reflects tissue/cell destruction and is associated with a wide range of disorders, including liver and lung disease. Interestingly, elevated LDH levels were previously shown to be associated with severe SARS infection [32]. The pathogenesis of COVID-19 has been also linked with the development of respiratory failure and hypoxia [2]. Hence there is convincing evidence linking high LDH levels with severe COVID-19 pneumonia and mortality [33,34,35]. Moreover, the normalization of serum LDH was consistently accurate in predicting treatment success of the patients [36].

LDH is an essential enzyme for anaerobic respiration, and its production has also been shown to be increased under hypoxic conditions in the liver [37]. Therefore, poor oxygen delivery can lead to hypoxic hepatitis, thus accounting for LDH elevation [38]. Hence, we detected no correlation between LDH and oxygen saturation. Still there is a consideration that all electronic records were obtained upon admission. Our results indicate that microcirculation disturbance exists from early stages of the disease and LDH levels could be regarded as a potential prognostic marker.

The liver is closely related to the synthesis of coagulation factors, which means that when the liver is damaged, it will directly affect the coagulation process. Furthermore, coagulation dysfunction in COVID-19 patients may cause liver damage due to thrombosis. Hence, the exact relationship between COVID-19-induced dysfunction of coagulation and liver damage is unclear [19,39]. In our study, we found significant positive correlation between elevated LDH and DD in 36.5% of the cases, as well as between LDH and fibrinogen in 15.2% of the cases. In 10.4% of the patients, AST showed significant correlation with elevated fibrinogen.

Another interesting and yet unpublished observation from our current study, was the significant increase AST/ALT (De Ritis ratio) in 75% of patients. The De Ritis ratio can also theoretically increase due to a reduction in AST clearance from the blood by the liver sinusoids [40]. This is supported by the fact that ACE2 occurrence has been also reported in endothelial cells [41]. However, the elevation of the AST/ALT ratio might also indicate a non-hepatic source of injury and massive death of non-liver cells, for example muscle damage [40]. Myalgia is a common symptom in 36% patients with COVID-19 [42]. Nonetheless, myalgia in patients with COVID-19 may be longer in duration than other viral infections and may be unresponsive to conventional painkillers. In addition to the classic mechanisms of myalgia known in viral infections, including generalized inflammation and cytokine response, COVID-19 can directly damage the musculoskeletal system due to the presence of ACE2 in skeletal muscles [43]. Consistently, viral damage in muscles—associated with increased LDH levels, in turn, promote an excessive accumulation of lactate, leading to muscle pain [44]. However, further studies need to confirm the molecular mechanisms and the cell-specific damage triggering an elevation of the De Ritis ratio.

Altogether, our results indicate that elevations in liver-related parameters are noticeably mild. It is tempting to speculate that abnormalities in liver function tests are not caused by direct SARS-CoV-2 liver injury, but very likely they are a result of “nonspecific reactive hepatitis”. Indeed, influenza infection as well as many other respiratory viruses produce similar elevations of liver function biomarkers, which is just a “collateral damage” related to immune interactions involving intrahepatic cytotoxic T and KCs [21,45]. Moreover, it is very likely that AST and LDH elevations do not necessarily arise from the liver alone, but can be also the results of myositis similar to that observed in influenza [21].

Since the liver is involved in drug metabolism, including nucleoside analogues and protease inhibitors, currently used for the treatment of COVID-19, drug-induced liver injury (DILI) must be considered during SARS-CoV-2 infection [46]. In our study, we exclusively used laboratory parameters electronically collected upon admission, in order to avoid any possible effects related to drug toxicity due to treatment. However, such effects were observed in many patients [8,13,14], who developed substantial increases in ALT and GGT levels during hospitalization Moreover, recent clinical trials using lopinavir and ritonavir in severe COVID-19 patients reported elevated levels of AST, ALT and total bilirubin as adverse side effects in a few patients [47]. Additionally, elevated levels of bilirubin and ALT/AST were also detected in patients treated with Remdesivir [48].

However, our study has several limitations. Although data were collected in a single center, being a guarantee of their homogeneity, the study was retrospective, and the data were extracted from electronic documentation. This potentially precluded the detailed analysis level done with a manual medical record review. Therefore, only the laboratory characteristics at admission were considered. Still some potential confounders could have been missing. Thus, we cannot exclude that some patients actually had LFTs abnormalities due to chronic liver diseases (CLD) before COVID-19. Hence, a recent large international registry study demonstrated that patients with CLD without cirrhosis appeared to have a similar risk of mortality following SARS-CoV-2 infection than those without CLD. However, patients with cirrhosis are at increased risk of death from COVID-19 [49].

Further research is urgently needed, in order to provide new insights in the multi-systemic effects of SARS-CoV-2 infection focusing on:

(i) Pathophysiological causes of fluctuation in liver injury parameters in COVID-19 patients;
(ii) A more detailed analysis of the effects of existing liver-related comorbidities on the outcome of the disease, and;
(iii) The potential mechanisms of hepatotoxicity of treatment for COVID-19. Detailed information from different national registries is particularly important in order to understand the heterogeneous character of COVID-19 and its real impact on the liver.

## Figures and Tables

**Table 1:** Characteristics of 799 patients infected with SARS-CoV-2, including demography and outcome, extracted from the electronic medical records.

**Characteristics**	
**Total number of patients**	799
**Sex, *n* (%)**	
Women	362 (45.3%)
Men	437 (54.7%)
**Age, years, *n* (%)**	
<19, *n* (%)	2 (0.3%)
20–29, *n* (%)	21 (2.6%)
30–39, *n* (%)	45 (5.6%)
40–49, *n* (%)	100 (12.5%)
50–59, *n* (%)	177 (22.2%)
60–69, *n* (%)	143 (17.9%)
70–79, *n* (%)	147 (18.4%)
80–89, *n* (%)	139 (17.4%)
90–99, *n* (%)	25 (3.1%)
**Outcome, *n* (%)**	
Dead	140 (17.5%)
Survived	659 (82.5%)

**Table 2:** Clinical characteristics of 799 patients with confirmed COVID-19, including gender differences, age and outcome of disease in connection to biomarkers of liver injury.

**PARAMETERS**	**ALT**	**AST**	**GGT**	**ALP**	**LDH**	**AST/ALT Ratio**
Total, $\bar{X}$ ± SD (U/L)	38.07 ± 39.61	43.57 ± 35.35	69.42 ± 85.11	87.32 ± 67.91	369.58 ± 159.08	1.41 ± 0.91
Normal, total *n* (%)	589 (74.27%)	461 (50.83%)	510 (65.38%)	573 (75.79%)	370 (44.16%)	193
Elevated, *n* (%)	204 (25.73%)	446 (49.17%)	270 (34.62%)	186 (24.21%)	400 (55.84%)	579
Women, $\bar{X}$ ± SD (U/L)	35.55 ± 2.39	41.67 ± 1.83	61.57 ± 4.59	88.59 ± 3.16	363.85 ± 8.90	1.46 ± 0.39
Men, $\bar{X}$ ± SD (U/L)	40.04 ± 1.85	45.52 ± 1.91	76.53 ± 4.37	86.00 ± 3.79	369.88 ± 7.77	1.33 ± 0.03
**AGE** **(years)**	**ALT** $\bar{X}$ **± SD (U/L)**	**AST** $\bar{X}$ **± SD (U/L)**	**GGT** $\bar{X}$ **± SD (U/L)**	**ALP** $\bar{X}$ **± SD (U/L)**	**LDH** $\bar{X}$ **± SD (U/L)**	**AST/ALT Ratio**
<19	251.500 ± 222.50	153.50 ± 114.50	272.50 ± 205.50	547.50 ± 140.50	838.50 ± 564.00	0.96 ± 0.39
20–29	47.95 ± 7.10	40.47 ± 4.71	68.74 ± 14.72	94.68 ± 12.62	311.37 ± 23.90	0.98 ± 0.09
30–39	52.20 ± 6.99	43.90 ± 4.72	82.63 ± 16.78	82.60 ± 6.99	307.88 ± 16.68	1.04 ± 0.07
40–49	46.21 ± 4.46	43.38 ± 4.28	78.65 ± 9.54	102.69 ± 13.34	342.90 ± 13.42	1.06 ± 0.04
50–59	45.54 ± 3.46	49.58 ± 3.81	84.13 ± 8.23	90.43 ± 4.52	382.29 ± 12.71	1.22 ± 0.03
60–69	35.54 ± 2.38	41.23 ± 1.96	74.64 ± 7.24	77.34 ± 4.22	345.32 ± 11.27	1.30 ± 0.04
70–79	30.17 ± 1.86	42.20 ± 2.37	60.95 ± 5.67	83.63 ± 5.25	394.28 ± 14.48	1.30 ± 0.04
80–89	29.46 ± 3.71	41.79 ± 3.18	51.91 ± 6.54	85.15 ± 5.09	380.51 ± 16.22	1.87 ± 0.15
90–99	19.50 ± 1.92	35.63 ± 3.77	36.69 ± 7.32	76.88 ± 5.27	349.84 ± 19.47	2.05 ± 0.14
**OUTCOME**	**ALT** $\bar{X}$ **± SD (U/L)**	**AST** $\bar{X}$ **± SD (U/L)**	**GGT** $\bar{X}$ **± SD (U/L)**	**ALP** $\bar{X}$ **± SD (U/L)**	**LDH** $\bar{X}$ **± SD (U/L)**	**AST/ALT Ratio**
Dead	33.59 ± 3.88	51.51 ± 4.71	64.43 ± 6.87	91.53 ± 5.97	439.72 ± 19.17	1.80 ± 0.07
Survived	38.88 ± 1.60	42.24 ± 1.29	70.80 ± 3.56	86.31 ± 2.77	352.73 ± 5.72	1.33 ± 0.04

ALT, alanine aminotransferase; AST, aspartate transaminase; ALP, alkaline phosphatase; GGT, gamma-glutamyltransferase; ALP, alkaline phosphatase; LDH, lactate dehydrogenase.

**Table 3:** Lineal correlation between liver tests and age.

	LINEAL CORRELATION TO AGE				
	Pearson Coefficient	*R* ^2^	*R*	*p* Value (Lineal Correlation)	% Cases Represented by Correlation
**ALT**	−0.234 *	0.055	0.234	0.0001	23.4%
**AST**	−0.072 *	0.005	0.072	0.046	7.2%
**GGT**	−0.151 *	0.023	0.151	0.0001	15.1%
**ALP**	−0.088 *	0.008	0.088	0.016	8.8%
**LDH**	0.073 ^†^	0.005	0.073	0.042	7.3%
**AST/ALT**	0.329 ^†^	0.108	0.329	0.0001	32.9%

Pearson coefficient, *R*^2^ value, *R* and *p* value related to correlation (calculated though ANOVA) are indicated. % of cases represented by each paired correlation is shown. Negative correlations are labeled with *. Positive correlations are marked with ^†^.

**Table 4:** Laboratory findings of COVID-19-positive patients on admission to hospital.

**PARAMETERS**	**SO_2_**	**FERRITIN**	**FIBRINOGEN**	**PT Time**	**D Dimers**
Total, $\bar{X}$ ± SD	95.45 ± 4.18%	1145.05 ± 1222.16 ng/mL	733.34 ± 209.13 mg/dL	17.54 ± 49.66 s	3160.68 ± 13,089.83 µg/L
**Normal, total *n*** **(%)**	694 (93.41%)	102 (18.85%)	20 (3.39%)	235 (30.36%)	46 (9.02%)
**Abnormal, *n*** **(%)**	49 (6.59%) (Decreased)	439 (81.15%) (Increased)	570 (96.61%) (Increased)	539 (69.64%) (Decreased)	464 (90.98%) (Increased)
**Women,** $\bar{X}$ **± SD (U/L)**	95.75 ± 0.21%	804.43 ± 53.69 ng/mL	695.63 ± 11.52 mg/dL	18.24 ± 3.33 s	3420.64 ± 850.14 µg/L
**Men,** $\bar{X}$ **± SD (U/L)**	95.19 ± 0.22%	1439.87 ± 82.57 ng/mL	763.25 ± 12.21 mg/dL	16.97 ± 1.77 s	2958.69 ± 791.39 µg/L
**AGE** **(years)**	**SO_2_ (%)**	**FERRITIN (ng/mL)**	**FIBRINOGEN (mg/dL)**	**PT Time (s)**	**D Dimers (µg/L)**
**<19**	95.50 ± 1.50	916.00 ± 823.00	537.00 ± 282.00	11.55 ± 0.05	3478.50 ± 2576.50
**20–29**	97.95 ± 0.41	734.20 ± 149.93	732.83 ± 70.06	12.96 ± 0.46	792.60 ± 140.35
**30–39**	96.97 ± 0.37	769.73 ± 209.10	634.26 ± 24.75	12.87 ± 0.17	470.63 ± 51.25
**40–49**	96.36 ± 0.30	1091.15 ± 165.12	784.40 ± 25.83	13.13 ± 0.14	1077.98 ± 451.12
**50–59**	96.24 ± 0.25	1309.02 ± 137.48	750.78 ± 17.60	13.44 ± 0.21	1515.38 ± 411.47
**60–69**	95.48 ± 0.33	1308.92 ± 111.77	766.80 ± 15.92	13.76 ± 0.41	3925.34 ± 1648.36
**70–79**	94.47 ± 0.41	1204.33 ± 100.18	749.51 ± 25.53	28.20 ± 9.19	5487.67 ± 2311.68
**80–89**	94.24 ± 0.44	903.75 ± 112.01	676.24 ± 15.96	19.84 ± 2.14	4873.30 ± 1719.06
**90–99**	94.91 ± 1.00	945.79 ± 196.733	664.33 ± 37.80	18.18 ± 1.78	4205.78 ± 1665.44
**OUTCOME**	**SO_2_ (%)**	**FERRITIN (ng/mL)**	**FIBRINOGEN (mg/dL)**	****PT Time (s)****	**D Dimers (µg/L)**
**Dead**	92.31 ± 0.57%	1629.05 ± 214.96 ng/mL	745.97 ± 19.26 mg/dL	17.54 ± 1.38 s	8082.74 ± 2570.75 µg/L
**Survived**	96.07 ± 0.13%	1081.26 ± 51.70 ng/mL	730.91 ± 9.58 mg/dL	17.54 ± 2.14 s	2285.39 ± 497.74 µg/L

SO_2_, oxygen saturation; PT, prothrombin.

**Table 5:** Lineal correlation of liver tests to O_2_ saturation and ferritin.

	**LINEAL CORRELATION TO O_2_ SATURATION**				
	**Pearson Coefficient**	***R*^2^**	***R***	***p* Value (Lineal Correlation)**	**% Cases Represented by Correlation**
**AST**	−0.072 *	0.005	0.072	0.054	7.2%
**LDH**	−0.228 *	0.052	0.228	0.0001	22.8%
**AST/ALT**	−0.106 *	0.011	0.106	0.005	10.6%
	**LINEAL CORRELATION TO FERRITIN**				
	**Pearson Coefficient**	***R*^2^**	***R***	***p*** **Value (Lineal Correlation)**	**% Cases Represented by Correlation**
**AST**	0.434 ^†^	0.188	0.434	0.0001	43.3%
**LDH**	0.357 ^†^	0.127	0.357	0.0001	35.7%
**AST/ALT**	−0.079 *	0.006	0.079	0.0001	7.9

Pearson coefficient, *R*^2^ value, *R* and *p* value related to correlation adjustment (calculated though ANOVA) are indicated. % of cases represented by each paired correlation is shown. Negative correlations are labeled with *. Positive correlations are marked with ^†^.

**Table 6:** Lineal correlation to D-dimers, fibrinogen and PT time to liver tests.

	**LINEAL CORRELATION TO D-DIMERS**				
	**Pearson Coefficient**	***R*^2^**	***R***	***p*** **Value (Lineal Correlation)**	**% Cases Represented by Correlation**
**AST**	0.076 ^†^	0.006	0.076	0.09	7.6%
**LDH**	0.365 ^†^	0.133	0.365	0.0001	36.5
**AST/ALT**	0.041 ^†^	0.002	0.041	0.361	4.1%
	**LINEAL CORRELATION TO FIBRINOGEN**				
	**Pearson Coefficient**	***R*^2^**	***R***	***p*** **Value (Lineal Correlation)**	**% Cases Represented by Correlation**
**AST**	0.013 ^†^	0.011	0.104	0.013	10.4%
**LDH**	0.152 ^†^	0.023	0.152	0.0001	15.2%
**AST/ALT**	−0.116 *	0.014	0.116	0.005	11.6%
	**LINEAL CORRELATION TO PT TIME**				
	**Pearson Coefficient**	***R*^2^**	***R***	***p* Value (Lineal Correlation)**	**% Cases Represented by Correlation**
**AST**	0.008 ^†^	0.0001	0.008	0.833	0.8%
**LDH**	0.035 ^†^	0.001	0.035	0.334	3.5%
**AST/ALT**	0.053 ^†^	0.003	0.053	0.145	5.3%

Pearson coefficient, *R*^2^ value, *R* and *p* value related to correlation adjustment (calculated though ANOVA) are indicated. % of cases represented by each paired correlation is shown. The negative correlations are labeled with *. Positive correlations are marked with ^†^.

## Data Availability

No new data were created or analyzed in this study. Data sharing is not applicable to this article.

## Supplementary Materials

The following are available online at https://www.mdpi.com/2077-0383/10/5/1039/s1, Table S1: Interaction studies between clinical characteristics, Table S2: Comparison of LFTs abnormalities detected at admission time in patients with SARS-CoV-2 in recently published studies.
