# Supplementary material for: Abnormal Liver Function Test in Patients Infected with Coronavirus (SARS-CoV-2): A Retrospective Single-Center Study from Spain
Source: J Clin Med. 2021 Mar 3;10(5):1039. doi: 10.3390/jcm10051039 (PMC7959465; doi:10.3390/jcm10051039)
Supplement: Supplementary file 1 [file jcm-10-01039-s001.pdf]

Supplementary. Table S1. Interaction studies between clinical characteristics and; (left) AST dependent variable; (center) LDH dependent variable; (right) AST/ALT ratio as dependent variable.

AST (Dependant variable)	*p*-Value
Gender vs. AST	0.509
Outcome vs. AST	0.0001
Age vs. AST	0.0001
Gender*Survival	0.425
Gender*Age	0.109
Survival*Age	0.0001
Gender*Survival*Age	0.002
**LDH (Dependant variable)**	***p*-Value**
Gender vs. LDH	0.317
Outcome vs. LDH	0.0001
Age vs. LDH	0.0001
Gender*Survival	0.015
Gender*Age	0.036
Survival*Age	0.0001
Gender*Survival*Age	0.0001
**AST/ALT (Dependant variable)**	***p*-Value**
Gender vs. AST/ALT	0.068
Outcome vs. AST/ALT	0.088
Age vs. AST/ALT	0.407
Gender*Survival	0.049
Gender*Age	0.990
Survival*Age	0.642
Gender*Survival*Age	0.845

Note: Two-way ANOVA was performed. P-value<0.05 indicates interaction between factors. Significant correlations are labelled in grey

Supplementary Table S2. Comparison of LFTs abnormalities detected at admission time in patients with SARS-CoV-2 in recently published studies.

Country	Study design	Time frame	Number of patients	LFTs abnormalities at admission (patients %)	LFTs abnormalities correlate with	Reference
Spain	single-center study (Hospital Universitario 12 de Octubre in Madrid)	25.02.20 - 23.04.20	799	ALT 25.73%, AST 49.17% GGT 34.62% ALP 24.21% LDH 55.84% AST/ALT 75%		Current study
France	multicenter study (CHU Montpellier, CH Narbonne).	10.03.20 - 18.04.20	234	Total 66.6%	-Risk of the ICU -Mortality	**[14]**
Italy	multicenter study (five internal medicine COVID-Units in two regions of Northern Italy)	22.02.20 - 8.04.20	565	Total 58% (AST 44%, ALT 32%, GGT 34%, ALP 5%)	-Risk of ICU -Mortality	**[12]**
Italy	single-center study (Fondazione Policlinico Universitario Agostino Gemelli IRCCS, Rome)	6.03.20 - 16.04.20	515	Total 31.3% (AST 20.4%, ALT 19%, GGT 13.6%)	-Risk of ICU	**[13]**
Austria	single-center study (University Hospital of Innsbruck, Austria)	26.02.20-21.04.20	655	AST 42%	-Acute phase proteins and IL-6	**[15]**
USA	multicenter study (12 hospitals in New York City, Long Island, and Westchester County, New York)	1.03.20-4.04.20	5700	AST 58.4%, ALT 39.0	-	**[10]**
China	single-centre Wuhan Jinyintan Hospital	1.01.20-20.01.20	99	AST 35% ALT 28% LDH 76%	-	**[3]**
China	multicenter study (Wuhan Jinyintan hospital and Union Hospital of Tongji Medical College)	20.12.19-23.01.20	81	AST 53%	-	**[21]**
China	multicenter study (seven designated tertiary hospitals in Zhejiang province)	10.01.20-26.01.20	62	AST 16.1% LDH 27%	-	**[22]**
China	single-centre (Yin-tan Hospital in Wuhan)	16.12.19-2.01.20	41	ALT 37% LDH 73%	-	**[4]**
China	single-centre (Third People's Hospital of Shenzhen)	11.01.20-21.02.20	417	Total 76,3% ALT 12.9% AST 18.23% GGT 16.3% ALP 4,82%	-progress to severe pneumonia (26.7%)	**[8]**
